# Distributed State Fusion Estimation of Multi-Source Localization Nonlinear Systems

**DOI:** 10.3390/s23020698

**Published:** 2023-01-07

**Authors:** Peng Liu, Shuyu Zhou, Peng Zhang, Mengwei Li

**Affiliations:** 1 Academy for Advanced Interdisciplinary Research, North University of China, Taiyuan 030051, China; 2 School of Instrument and Intelligence, North University of China, Taiyuan 030051, China; 3 School of Instrumentation and Electronic, North University of China, Taiyuan 030051, China

**Keywords:** multi-source localization, unknown and bounded noise, dual set-membership filtering, distributed fusion strategy

## Abstract

For the state estimation problem of a multi-source localization nonlinear system with unknown and bounded noise, a distributed sequential ellipsoidal intersection fusion estimation algorithm based on the dual set-membership filtering method is proposed to ensure the reliability of the localization system. First, noise with unknown and bounded characteristics is modeled by using bounded ellipsoidal regions. At the same time, local estimators are designed at the sensor link nodes to filter out the noise interference in the localization system. The local estimator is designed using the dual set-membership filtering algorithm. It uses the dual principle to find the minimizing ellipsoid that can contain the nonlinear function by solving the optimization problem with semi-infinite constraints, and a first-order conditional gradient algorithm is used to solve the optimization problem with a low computational complexity. Meanwhile, the communication confusion among multiple sensors causes the problem of unknown correlation. The obtained estimates of local filters are fused at the fusion center by designing a distributed sequential ellipsoid intersection fusion estimation algorithm to obtain more accurate fusion localization results with lower computational cost. Finally, the stability and reliability of the proposed distributed fusion algorithm are verified by designing a simulation example of a multi-source nonlinear system.

## 1. Introduction

Multi-source data fusion is widely used as a hot research problem in vehicle localization [1,2], autonomous driving [3,4], and artificial intelligence [5,6]. It removes the drawback of not providing more data information by a single sensor and considers multiple sensor data samples for weight fusion to obtain more accurate data information [7]. This is well reflected in the localization system. In order to obtain more accurate positioning results, people provide more information for vehicle positioning by adding different kinds of sensors [8]. However, how to effectively use the information provided by multiple source sensors to ensure more reliable positioning results is worth deep consideration.

Currently, the existing methods for multi-sensor data fusion estimation are divided into centralized fusion and distributed fusion. The advantage of centralized fusion is that it can provide optimal fusion information. However, it can lead to inaccurate fusion information because the multi-source system may fail and damage individual sensors in actual operation. However, the distributed fusion strategy solves this problem. It processes multiple sensor measurement processes in parallel, which can well isolate the faulty sensors [9]. The distributed fusion strategy ensures the reliability of the system fusion results. It is widely used in positioning systems. In reference [10], the proposed distributed Kalman filtering method was used in a cooperative localization system with integrated measurements. In addition, the distributed fusion strategy has been applied in cooperative sensing based on vision transformers for vehicle networking [11] and cooperative positioning for autonomous vehicle safety [12]. These improve the reliable and flexible performance of vehicle positioning systems. Therefore, to ensure the reliability and flexibility of multi-source localization systems, this paper uses a distributed fusion strategy to obtain more reliable fusion information.

### 1.1. Related Work

For multi-source localization systems, a common model of localization systems is the idealized linear system. However, in the actual positioning process, the input and output of the system can be disproportionate due to many factors that are not under control in the system [13]. In order to more realistically consider the actual situation in the target localization process, it is more realistic and reliable to use a nonlinear system to describe the state space model of the target localization system [14]. At the same time, the system noise of the positioning system is usually described using the exact distribution. The existing Bayesian filtering methods are the unscented Kalman filtering (UKF) method, the extended Kalman filtering (EKF) method, and so on. They all can obtain good filtering effects. However, in the actual localization process, we cannot describe the statistical characteristics of the system noise accurately. If the existing Bayesian filtering strategy is chosen, the accuracy of the localization results may be reduced. In order to describe the system noise during localization, an unknown and bounded (UAD) ellipsoidal region was used to describe the system noise in reference [15]. Therefore, how to design a suitable localization filter for a multi-source nonlinear system with UAD noise disturbances is one of the focuses of the discussion in this paper.

An existing filtering method for UAD noise disturbances in the system is the set-membership filtering (SMF) strategy [16]. As the noise characteristics are characterized by a bounded range, the SMF works by finding a minimum estimated ellipsoid to contain the possible range of the target’s state [17]. However, it is difficult to apply the SMF to nonlinear systems with UAD noise. In [18], the extended set-membership filter (ESMF) was proposed. It works by performing a Taylor expansion on the nonlinear function in the system and then qualifying each expansion. However, it is difficult to define mathematically due to the presence of higher-order residual terms [19]. If the higher-order terms are discarded, it will affect the accuracy of the estimation results. Therefore, the problem of solving a semi-infinite programming problem to describe the linearization of a nonlinear function was proposed [20]. It uses the dual principle to transform the semi-infinite programming problem to find a minimizing ellipsoid that can contain the nonlinear function [21]. The nonlinear function is linearized by using the information related to the minimizing ellipsoid [22]. The integrity of the information after the transformation of the nonlinear function is ensured. Based on the above analysis, this paper uses the dual principle to design a dual set-membership (DSM) local filter at each sensor link node to achieve noise filtering [23]. Meanwhile, the computational cost is increased when dealing with optimization problems. This paper uses a first-order conditional gradient algorithm to quickly solve the problem of finding the minimizing ellipsoid [24,25]. A guarantee is provided for the local estimator to obtain a minimum updated ellipsoid of the state estimate. For the multi-sensor distributed system, it is important to design a high-performance fusion estimator to fuse the local estimates in order to make full use of the estimation information provided by multiple sensors.

For the problem of data fusion in multi-sensor distributed systems, the existing fusion techniques are *Minkowski* sum fusion strategies. They use a joint fusion approach to improve the reliable performance of the system. An intersection method to find the maximized global estimated ellipsoid is proposed [26], which provides accurate fusion results for multi-source systems. The key to data fusion is to extract the relevant information provided by different sensors in a representational way. It obtains fused information with higher accuracy through a fusion strategy. However, in the actual localization process, there may be a mix of communications between multiple sensors. This results in a situation where the inter-sensor correlation information is unknown, which brings unnecessary difficulties to the fusion process [27]. For the processing of unknown correlations between sensors, a covariance intersection (CI) fusion method was proposed [28]. This method directly fuses the locally estimated information and finds the minimized ellipsoid containing the intersecting region by means of an optimization problem [29]. As this method discards the consideration of correlations between local estimates, the CI method leads to the absence of qualifications in the optimization problem. It causes the problem of conservative estimation results [30]. In order to obtain the fusion results with higher accuracy, the ellipsoidal intersection (EI) fusion method was proposed [31]. It is based on the principle of explicitly describing the unknown correlation between sensors using information from local estimation. By parameterizing the fusion formulation, the minimum ellipsoid containing the correlation information between the local estimates was found. In contrast, when the case of unknown correlation between sensors occurs [32], the EI fusion method can ensure that the fusion results obtain higher accuracy. This method describes the optimization problem arising in the fusion process with explicit expressions. It greatly saves the computational cost of the system [33].

### 1.2. Paper Contributions

**Figure 1:**
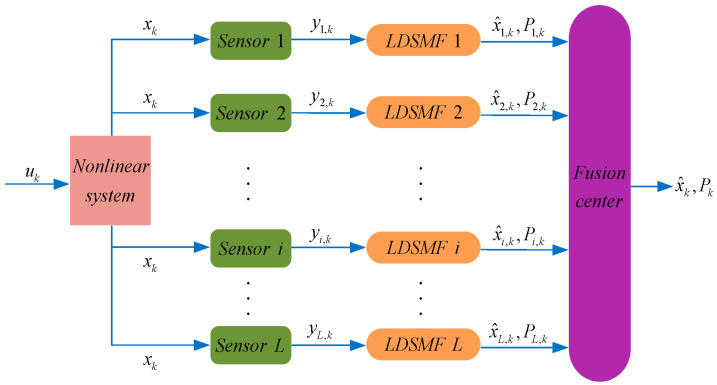
The architecture diagram of DSEI fusion positioning system.

This paper studies the data fusion problem for a multi-source localization nonlinear system with UAD noise interference. In order to obtain a flexible and accurate localization system, we use a distributed fusion strategy for the design of the multi-source localization system. To describe the system noise in the localization process more realistically, we use a bounded ellipsoidal region to model the design of the UAD system noise. Meanwhile, we describe the process of linearizing the nonlinear function in terms of solving a semi-infinite constrained optimization problem based on the dual principle. The local estimator is designed at the sensor link nodes. It is based on the idea of combining the set-membership principle with the dual principle to design a DSM filter to filter out the interference of system noise. A first-order conditional gradient algorithm is used to reduce the computational complexity of solving the filtering process. Finally, we resolve the problem of unknown correlation arising from the communication chaos between multiple source sensors. A distributed sequential ellipsoidal intersection (DSEI) fusion estimation algorithm is designed to fuse the obtained local filtering estimates at the fusion center. The architecture diagram of the DSEI fusion positioning system is shown in Figure 1. The simulation example verifies that the selected EI fusion strategy can obtain more accurate fusion localization results at a lower computational cost. 

## 2. Problem Description

For the verification of the target tracking performance of a positioning system, the accuracy of the positioning results is one of the primary conditions to be considered. The common positioning system model is an idealized linear system. The actual situation of the positioning system is more realistically considered in the target tracking process. This paper adopts a nonlinear system to describe the state space model of the target positioning system. It provides a guarantee for obtaining more realistic and reliable positioning results [2]. The state space model of the nonlinear positioning system is as follows:

(1) $$x_{k+1}=f_{k}(x_{k},u_{k})+w_{k}$$

where $x_{k}\in R^{n}$ denotes the target tracking state vector at the kth moment and $u_{k}$ denotes the external control input vector. $f_{k}(x_{k},u_{k})$ is a nonlinear function in the state equation and it is continuously differentiable. $w_{k}$ is the additive process noise in the state equation.

For the actual localization process, we cannot describe the statistical characteristics of the noise accurately. If an idealized model with an exact distribution is used to model the noise, the accuracy of the localization results may be reduced [12]. In order to truly reflect the working performance of the positioning system in the actual process, the noise is modeled by designing an UAD set of ellipsoidal regions [15]. That is, the process noise $w_{k}$ exists in a bounded ellipsoid $W_{k}$.

(2) $$w_{k}\in W_{k}=\left\{w_{k}:w_{k}^{T}Q_{k}^{-1}w_{k}\leq 1\right\}$$

where the ellipsoid $W_{k}$ is the shape matrix of $Q_{k}$, which is a positive definite matrix with known and appropriate dimensions.

In order to obtain more accurate positioning results, this paper designs a high-precision positioning system using multiple sensors. The measurement model of the sensors in the positioning system is as follows:

(3) $$y_{i,k}=h_{i,k}(x_{k})+v_{i,k},i=1,2,\cdots ,L$$

where $y_{i,k}\in R^{m_{i}}$ denotes the observation of the nonlinear positioning system by the *i*th sensor at the *k*th moment. The measurement function $h_{i,k}(x_{k})$ is described by a continuously differentiable nonlinear function. At the same time, the characteristics of the measurement noise $v_{i,k}$ are assumed to be an UAD region. It exists in a bounded ellipsoid $V_{i,k}$.

(4) $$v_{i,k}\in V_{i,k}=\left\{v_{i,k}:v_{i,k}^{T}P_{k}^{-1}v_{i,k}\leq 1\right\}$$

As the system noise is modeled with an UAD ellipsoidal region, we assume that the initial target state $x_{0}$ is contained in a known bounded ellipsoid $\varepsilon_{0}$.

(5) $$\varepsilon_{0}=\left\{x\in R^{n}:{\left(x-{\hat{x}}_{0}\right)}^{T}P_{0}^{-1}\left(x-{\hat{x}}_{0}\right)\leq 1\right\}$$

where ${\hat{x}}_{0}$ is the center of the ellipsoid $\varepsilon_{0}$. $P_{0}$ is the shape matrix of the ellipsoid $\varepsilon_{0}$ and it is known and positive definite.

For filtering methods of nonlinear systems, the extended filtering technique is often used. It is based on the principle that the nonlinear function is linearized by Taylor expansion [18]. The Taylor expansion of the nonlinear function results in the following.

(6) $$\begin{array}{ll} x_{k+1}= & f_{k}\left(x_{k}^{o},u_{k}^{o}\right)+\frac{\partial f_{k}\left(x_{k}^{o},u_{k}\right)}{\partial x}\left(x_{k}-x_{k}^{o}\right)\\ & +\frac{\partial f_{k}\left(x_{k},u_{k}^{o}\right)}{\partial u}\left(u_{k}-u_{k}^{o}\right)+R_{k}^{f}\left(x_{k},x_{k}^{o},u_{k}^{o}\right)+w_{k} \end{array}$$

where $R_{k}^{f}\left(x_{k},x_{k}^{o},u_{k}^{o}\right)$ is the higher-order Lagrange’s remainder.

As the system noise characteristics are UAD, the set-membership filtering strategy is given primary consideration [18]. It encompasses the possible range of target states by finding a minimum estimated ellipsoid. However, the characteristics of the localization system are nonlinear. When the set-membership filtering method is used to filter the noise, due to the presence of higher-order residual terms $R_{k}^{f}\left(x_{k},x_{k}^{o},u_{k}^{o}\right)$ after Taylor expansion, it is difficult to compute the Hessian matrix consisting of the second-order partial derivatives of the nonlinear functions. If the existing ESMF method is used, a large ellipsoidal region is required to characterize the range of state estimates. This would only lead to overly conservative estimation results. Clearly, this approach is an unwise choice for noise filtering of nonlinear systems with UAD noise. Although the higher-order residual term can also be described by other terms, it is still difficult to find a tight ellipsoid to define $R_{k}^{f}\left(x_{k},x_{k}^{o},u_{k}^{o}\right)$ [19].

Based on the above analysis, we urgently need to find a new set-membership filter to eliminate the interference with UAD noise in the nonlinear system, to lay a solid foundation for obtaining a high-accuracy localization result.

## 3. Local Estimator Based on DSM Filtering Method

In this section, for the presence of UAD noise disturbances in a multi-source localization nonlinear system, a DSM local filter is proposed to effectively filter out the system noise disturbances. First, we assume that the ellipsoidal region $\varepsilon_{k}$ containing all possible values of the target state $x_{k}$ at the kth moment has been obtained.

(7) $$\begin{array}{l} \varepsilon_{i,k}=\left\{x\in R^{n}:{\left(x-{\hat{x}}_{i,k}\right)}^{T}P_{i,k}^{-1}\left(x-{\hat{x}}_{i,k}\right)\leq 1\right\}\\ =\left\{x:x={\hat{x}}_{i,k}+I_{i,k}\varsigma_{i,k},P_{i,k}=I_{i,k}I_{i,k}^{T},\left\|\varsigma_{i,k}\right\|\leq 1\right\} \end{array}$$

where ${\hat{x}}_{i,k}$ denotes the center of the ellipsoid $\varepsilon_{i,k}$; $P_{i,k}$ indicates that the shape matrix of the ellipsoid $\varepsilon_{i,k}$ has appropriate dimensions and positive definite properties.

Due to the existence of the characteristics of nonlinear systems, the region $F_{i,k}$ after the transformation of the target state $x_{i,k}$ by a nonlinear function is irregular [20]. If the ESMF approach is used to design the filter, it will cause the problem of conservative estimation. As the system noise is characterized by a bounded region, can we find a minimum ellipsoid containing the set $F_{i,k}$? The nonlinear function is described as a linear function with the help of the information related to the minimum ellipsoid.

### 3.1. Dual Strategy Based on First-Order Gradient Algorithm

Based on the above analysis, we find a minimal ellipsoid that can contain a nonlinear function. The solution of the minimax ellipsoid is often handled using optimization techniques. As the target state values are not unique, the set $F_{i,k}$ containing the nonlinear functions is a bounded region. The conditional constraints of the minimizing ellipsoid containing the bounded region have infinite numbers. For optimization problems with an infinite number of constraints, we call them semi-infinite programming problems. In [21], it proposes a dual-based problem to solve the semi-infinite programming problem and proves that the dual principle can effectively solve the semi-infinite programming problem.

However, we use the dual approach to solve the semi-infinite programming problem that arises in the process of finding the minimizing ellipsoid. First, we compute the target state values through continuously differentiable nonlinear functions, which is obtained as an irregular compact ensemble $F_{i,k}=\left\{f_{k}\left(x_{k},u_{k}\right):x_{k}\in \varepsilon_{i,k}\right\}$ [23]. Our goal is to find a minimal ellipsoid $\varepsilon_{i,f_{k}}$ that can contain the set $F_{i,k}$. The minimax ellipsoid $\varepsilon_{i,f_{k}}$ is solved as follows:

$$\underset{P_{i,f_{k}},{\hat{x}}_{i,f_{k}}}{\min}\log\det\left(P_{i,f_{k}}\right)$$

$$s.t.\;{\left[x_{i,f_{k}}-{\hat{x}}_{i,f_{k}}\right]}^{T}P_{i,f_{k}}^{-1}\left[x_{i,f_{k}}-{\hat{x}}_{i,f_{k}}\right]\leq 1$$

(8) $$\forall \;x_{i,f_{k}}\in F_{i,k}$$

where ${\hat{x}}_{i,f_{k}}$ denotes the center of the ellipsoid $\varepsilon_{i,f_{k}}$. $P_{i,f_{k}}$ indicates the shape matrix of the ellipsoid $\varepsilon_{i,f_{k}}$. The general expression for the optimal solution of the semi-infinite programming problem (8) can be expressed as:

(9) $${\hat{x}}_{i,f_{k}}^{*}=\int_{F_{i,k}}x\;d\mu^{*},\;P_{i,f_{k}}^{*}=\int_{F_{i,k}}xx^{T}d\mu^{*}-{\hat{x}}_{i,f_{k}}^{*}{\hat{x}}_{i,f_{k}}^{*}{}^{T}$$

Our goal is to obtain the optimal solution of the semi-infinite programming problem. We use the dual principle to convert the problem of solving the minimizing ellipsoid into the corresponding maximizing optimization problem.

$$\underset{\mu }{\max}\log\det\left(\int_{F_{i,k}\times \left\{1\right\}}\tilde{x}{\tilde{x}}^{T}d\mu \right)$$

(10) $$s.t.\;\int_{F_{i,k}\times \left\{1\right\}}d\mu =1,\;\mu \geq 0$$

where $\tilde{x}$ denotes the device of the vector $\left[\begin{array}{cc} x^{T} & 1 \end{array}\right]$.

As the measure of the constraint in the optimization problem can be described in the form of a discrete metric [22], we can randomly select a set of points ${\tilde{x}}_{j}$ from the set $F_{i,k}\times \left\{1\right\}$ and apply them to the optimization problem. By discretizing the continuous constraints, the optimization problem with discrete constraints is expressed as:

$$\underset{\mu_{j}}{\max}\log\det\left(\sum_{j=1}^{m}\mu_{j}{\tilde{x}}_{j}{\tilde{x}}_{j}^{T}\right)$$

(11) $$s.t.\;\sum_{j=1}^{m}\mu_{j}=1,\mu_{j}\geq 0$$

We convert the semi-infinite constrained problem of solving the minimum ellipsoid into a semi-positive definite planning problem with discrete constraints. A first-order conditional gradient algorithm is proposed for solving the semi-positive definite optimization problem [23]. This is a projection-free method. It removes the computation of the Hessian matrix consisting of second-order partial derivatives by performing a first-order linear approximation to the objective function. The computational cost of the iteration of the first-order conditional gradient algorithm is $O\left(n^{2}+nm\right)$, which is much smaller than the computational cost of $O\left(n^{4.75}m^{1.5}\right)$ using the interior point method [24]. Therefore, the first-order conditional gradient algorithm can reduce the computational complexity of the solution process of the semi-positive definite optimization problem.

We use the first-order conditional gradient algorithm to solve the semi-positive definite optimization problem. First, let the objective function $G\left(\mu \right)=\log\det\left(\sum_{j=1}^{m}\mu_{j}{\tilde{x}}_{j}{\tilde{x}}_{j}^{T}\right)$. Then, the objective function is expanded at $\mu_{o}$. The objective function of the first-order approximation is as follows:

(12) $$G\left(\mu \right)\approx G\left(\mu_{o}\right)+\partial G\left(\mu_{o}\right)\left(\mu -\mu_{o}\right)$$

Substituting (12) into the optimization problem (11), it is obtained that

$$\underset{\mu_{i}}{\max}\left(G\left(\mu_{o}\right)+\partial G\left(\mu_{o}\right)\left(\mu -\mu_{o}\right)\right)$$

(13) $$s.t.\;\sum_{j=1}^{m}\mu_{j}=1,\mu_{j}\geq 0$$

When $\partial G\left(\mu_{o}\right)\left(\mu_{j}^{*}-\mu_{o}\right)=0$, $\mu_{o}$ is the K-T point of the above optimization problem. When $\partial G\left(\mu_{o}\right)\left(\mu_{j}^{*}-\mu_{o}\right)\neq 0$, the vector $\left(\mu_{j}^{*}-\mu_{o}\right)$ denotes the direction of descent of the objective function at the point $\mu_{o}$. If the above two points are satisfied, we have a finite optimal solution $\mu_{j}^{*}$. At the same time, we can obtain the information about the center ${\hat{x}}_{i,f_{k}}$ and the shape matrix $P_{i,f_{k}}$ of the minimum ellipsoid $\varepsilon_{i,f_{k}}$.

$${\hat{x}}_{i,f_{k}}=\sum_{j=1}^{m}\mu_{j}^{*}x_{j},P_{i,f_{k}}=\sum_{j=1}^{m}\mu_{j}^{*}x_{j}x_{j}^{T}-{\hat{x}}_{f_{k}}{\hat{x}}_{f_{k}}{}^{T}$$

Immediately afterward, the minimum ellipsoid $\varepsilon_{i,f_{k}}$ can be obtained.

(14) $$\varepsilon_{i,f_{k}}=\left\{\begin{array}{l} x:x={\hat{x}}_{i,f_{k}}+I_{i,f_{k}}\varsigma_{i,f_{k}}\\ \left\|\varsigma_{i,f_{k}}\right\|\leq 1,I_{i,f_{k}}I_{i,f_{k}}^{T}=P_{i,f_{k}} \end{array}\right\}$$

Using the information about the minimum ellipsoid $\varepsilon_{i,f_{k}}$, we can convert the nonlinear function into a linearized linear function.

(15) $$x_{k+1}=f_{k}\left(x_{k},u_{k}\right)+\omega_{k}={\hat{x}}_{i,f_{k}}+E_{i,f_{k}}I_{i,f_{k}}+\omega_{k}$$

Based on the above analysis of the transformation of nonlinear functions, we transform the measurement transfer function in the system. First, we describe the target state values using the information in the measurement equation (Equation (3)).

(16) $$P_{i,x}x_{i,k+1}=h_{{}_{i,k+1}}^{-1}(y_{i,k+1}-v_{i,k+1})$$

where $P_{i,x}$ denotes the transformation projection matrix. Based on the optimization technique, we can bound $P_{i,x}x_{i,k+1}$ by finding a minimum measurement ellipsoid $\varepsilon_{i,k+1}^{z}$. The process of determining the minimum measurement ellipsoid $\varepsilon_{i,k+1}^{z}$ is also a programming problem with an infinite number of constraints.

$$\underset{P_{i,k+1}^{z},{\hat{x}}_{i,k+1}^{z}}{\min}\log\det\left(P_{i,k+1}^{z}\right)$$

$$s.t.\;{\left[x_{i,k+1}^{z}-{\hat{x}}_{i,k+1}^{z}\right]}^{T}{\left(P_{i,k+1}^{z}\right)}^{-1}\left[x_{i,k+1}^{z}-{\hat{x}}_{i,k+1}^{z}\right]\leq 1$$

(17) $$\forall \;x_{i,k+1}^{z}\in C_{i,k+1}$$

Finally, based on the first-order conditional gradient algorithm principle, we can obtain the optimal solution to the semi-infinite constrained problem. By substituting the optimal solution into Equation (17), the result of the linearized measurement equation is obtained as follows:

(18) $$P_{i,x}x_{i,k+1}={\hat{x}}_{i,k+1}^{z}+I_{i,k+1}^{z},\;{\left(I_{i,k+1}^{z}\right)}^{T}{\left(P_{i,k+1}^{z}\right)}^{-1}I_{i,k+1}^{z}\leq 1$$

In this way, we convert the nonlinear system into a linear system.

### 3.2. Design of Local DSM Filters

Due to the interference of UAD noise in the system, this paper uses the DSM principle for the design of the local filter. The DSM filtering method is to find a minimum estimated ellipsoid to contain the possible range of target states. Therefore, we discuss the prediction step and update step based on the information of the minimizing ellipsoid $\varepsilon_{i,k+1}^{z}$ and $\varepsilon_{i,f_{k}}$.

**Figure 2:**
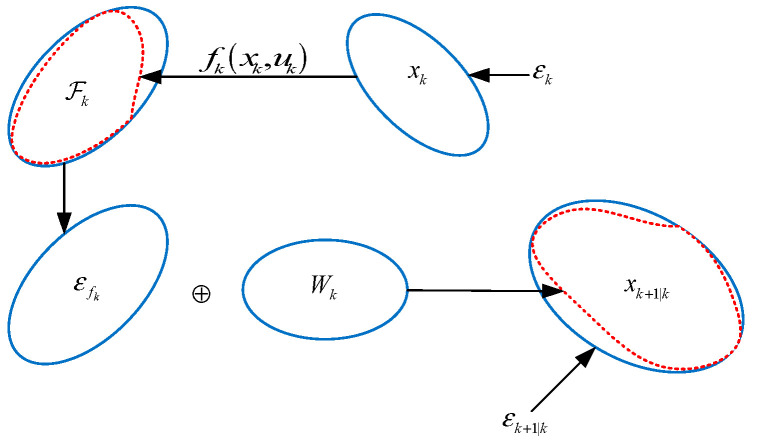
The process of determining the prediction ellipsoid $\varepsilon_{i,k+1|k}$.

However, the target state $x_{i,k+1}$ is determined by the state transition function $f_{k}\left(x_{k},u_{k}\right)$ and the process noise $\omega_{k}$. Thus, the target state value $x_{i,k+1}$ exists in the *Minkowski* sum of the ellipsoid $\varepsilon_{i,f_{k}}$ and the process noise $W_{k}$. For the solution of the *Minkowski* sum, we can determine the range of $\varepsilon_{i,f_{k}}⊕W_{k}$ by finding a minimizing ellipsoid [21]. Therefore, the design of the prediction step is the process of finding the minimizing ellipsoid containing $\varepsilon_{i,f_{k}}⊕W_{k}$. The process of determining the prediction ellipsoid $\varepsilon_{k+1|k}^{i}$ is shown in Figure 2. The center and shape matrices of the predicted ellipsoid $\varepsilon_{k+1|k}^{i}$ are as follows:

$${\hat{x}}_{k+1|k}^{i}={\hat{x}}_{i,f_{k}}$$

(19) $$P_{k+1|k}^{i}\left(\sigma_{k}\right)=\left(1+\sigma_{k}^{-1}\right)P_{i,f_{k}}+\left(1+\sigma_{k}\right)Q_{k}$$

where $\sigma_{k}$ satisfies:

$$\underset{\sigma_{k}>0}{\min}r\left(P_{k+1|k}^{i}\left(\sigma_{k}\right)\right)$$

As the minimum ellipsoid containing $\varepsilon_{i,f_{k}}⊕W_{k}$ is unique, the optimal value of $\sigma_{k}^{*}$ can be obtained: $\sqrt{tr\left(P_{i,f_{k}}\right)}/\sqrt{tr\left(Q_{k}\right)}$. The minimum prediction ellipsoid $\varepsilon_{k+1|k}^{i}$ is obtained by substituting the optimal solution into Equation (19).

.

$${\hat{x}}_{k+1|k}^{i}={\hat{x}}_{f_{k}}$$

(20) $$P_{k+1|k}^{i}=\left(1+\frac{\sqrt{tr\left(Q_{k}\right)}}{\sqrt{tr\left(P_{i,f_{k}}\right)}}\right)P_{f_{k}}+\left(1+\frac{\sqrt{tr\left(P_{i,f_{k}}\right)}}{\sqrt{tr\left(Q_{k}\right)}}\right)Q_{k}$$

Similarly, for the design of the state update step, we determine the update ellipsoid $\varepsilon_{i,k+1}$ using the intersection part of the minimum prediction ellipsoid $\varepsilon_{k+1|k}^{i}$ and the measurement ellipsoid $\varepsilon_{i,k+1}^{z}$. The center and shape matrix of the measurement update ellipsoid $\varepsilon_{i,k+1}$ are shown as follows:

$$\begin{array}{ll} {\hat{x}}_{i,k+1} & ={\hat{x}}_{k+1|k}^{i}+\frac{P_{k+1|k}^{i}}{1-\rho_{i,k+1}}P_{i,x}^{T}\\ & \cdot {\left[P_{i,x}\frac{P_{k+1|k}^{i}}{1-\rho_{i,k+1}}P_{i,x}^{T}+\frac{P_{i,k+1}^{z}}{\rho_{i.k+1}}\right]}^{-1}\\ & \cdot \left(x_{i,k+1}^{z}-P_{i,x}{\hat{x}}_{k+1|k}^{i}\right) \end{array}$$

$$P_{i,k+1}=\left(1-\delta_{i,k+1}\right){\bar{P}}_{i,k+1},\;0\leq \rho_{i,k+1}\leq 1$$

$${\bar{P}}_{i,k+1}={\left[\left(1-\rho_{i,k+1}\right){\left(P_{k+1|k}^{i}\right)}^{-1}+\rho_{i,k+1}P_{i,x}^{T}{\left(P_{i,k+1}^{z}\right)}^{-1}P_{i,x}\right]}^{-1}$$

(21) $$\begin{array}{ll} \delta_{i,k+1} & ={\left({\hat{x}}_{i,k+1}^{z}-P_{i,x}{\hat{x}}_{k+1|k}^{i}\right)}^{T}\\ & \cdot {\left[P_{i,x}\frac{P_{k+1|k}^{i}}{1-\rho_{i,k+1}}P_{i,x}^{T}+\frac{P_{i,k+1}^{z}}{\rho_{i,k+1}}\right]}^{-1}\\ & \cdot \left({\hat{x}}_{i,k+1}^{z}-P_{i,x}{\hat{x}}_{k+1|k}^{i}\right) \end{array}$$

where $\rho_{i,k+1}$ satisfies:

$$\min\;r\left(P_{i,k+1}^{z}\right)$$

(22) $$s.t.\;0\leq \rho_{i,k+1}\leq 1$$

Based on the above analysis, we have completed the design of a local filter. It filters out the UAD noise disturbances present in the multi-source nonlinear system by the DSM filtering method. The proposed filtering method reduces the computational cost of the localization system to a certain extent.

## 4. Distributed Ellipsoidal Intersection Fusion Estimation

For a multi-source localization system, in order to effectively deal with unexpected situations that affect the measurement results such as the failure or malfunction of a sensor, a distributed system is used to improve the reliability and flexibility of the multi-source positioning system. Multiple sensors form a distributed positioning network to communicate through a wireless sensor network. These sensors can be placed at any position of the vehicle to ensure the flexibility of the system and the reliability of the measurement results [25]. However, multi-sensor systems may suffer from communication confusion during practical applications. The communication confusion can lead to undetermined correlation information between sensors. It brings some difficulties to the design of fusion estimation. In order to effectively deal with the occurrence of unknown correlation situations between sensors, we analyze the existing distributed fusion estimation algorithms to find a fusion estimation method that can solve the unknown correlation problem for the design of multi-source localization systems.

The unknown correlation means that the mutual correlation covariance $\mathrm{cov}\left(x_{i},x_{j}\right)$ between sensors is unknown [27]. For the treatment of unknown correlations, it proposes a fusion method to avoid solving the mutual correlation covariance [28]: the fusion method of the covariance intersection (CI). As the correlation information between sensors is difficult to determine, the CI method directly fuses the locally estimated information, and it finds the minimized ellipsoid containing the intersecting regions by means of an optimization problem [29]. This method is easy to operate and is often used [30]. The CI fusion method is as follows:

$${\hat{x}}_{CI}=P_{CI}\left[\beta P_{i}^{-1}{\hat{x}}_{i}+\left(1-\beta \right)P_{j}^{-1}{\hat{x}}_{j}\right]$$

(23) $$P_{CI}={\left[\beta P_{i}^{-1}+\left(1-\beta \right)P_{j}^{-1}\right]}^{-1}$$

where the optimal weight value β satisfies:

(24) $$\begin{array}{l} \underset{\beta }{\min}tr\left(P_{CI}\right)\\ s.t.\;0\leq \beta \leq 1 \end{array}$$

However, as the CI fusion process discards the analysis of unknown correlations, the CI fusion method needs to provide a large range of fusion results. It leads to the absence of qualifying conditions in the optimization problem. If the CI method is used for the design of distributed fusion estimators. It leads to the problem of conservative estimation of the localization results, which reduces the accuracy of the localization results of the multi-source localization system [31]. In order to obtain accurate fusion information, we need to take full consideration of the unknown correlation information between sensors.

For dealing with the problem of unknown correlations, a new fusion method is proposed: the ellipsoidal intersection (EI) fusion method [32]. It is based on the principle of explicitly describing the unknown correlations between sensors using information from local estimation. It then parameterizes the fusion formulation. The EI fusion algorithm has the advantage of computational complexity. As it describes the process of finding the minimum ellipsoid with an explicit expression, the EI fusion algorithm saves the computational cost during the operation of the algorithm. This method avoids the problem of conservative estimation in CI fusion methods. It can guarantee the accuracy of fusion results and reduce the computational complexity of the system [33]. Thus, the EI fusion method is used to design the fusion estimator. The EI fusion method is as follows:

$${\hat{x}}_{EI}=P_{EI}\left(P_{i}^{-1}{\hat{x}}_{i}+P_{j}^{-1}{\hat{x}}_{j}-\Gamma^{-1}\gamma \right)$$

(25) $$P_{EI}={\left(P_{i}^{-1}+P_{j}^{-1}-\Gamma^{-1}\right)}^{-1}$$

where the correlation means γ and variances Γ are expressed through the $x_{i}$ and $x_{j}$ information.

$$\Gamma =S_{i}\sqrt{D_{i}}S_{j}D_{\Gamma }S_{j}^{-1}\sqrt{D_{i}}S_{i}^{-1}$$

(26) $$\begin{array}{ll} \gamma = & {\left(P_{i}^{-1}+P_{j}^{-1}-2\Gamma^{-1}+2\eta I\right)}^{-1}\\ & \times \left(\begin{array}{l} \left(P_{j}^{-1}-\Gamma^{-1}+\eta I\right){\hat{x}}_{i}\\ +\left(P_{i}^{-1}-\Gamma^{-1}+\eta I\right){\hat{x}}_{j} \end{array}\right) \end{array}$$

where S denotes the feature vector matrix and D denotes the feature diagonal matrix.

**Figure 3:**
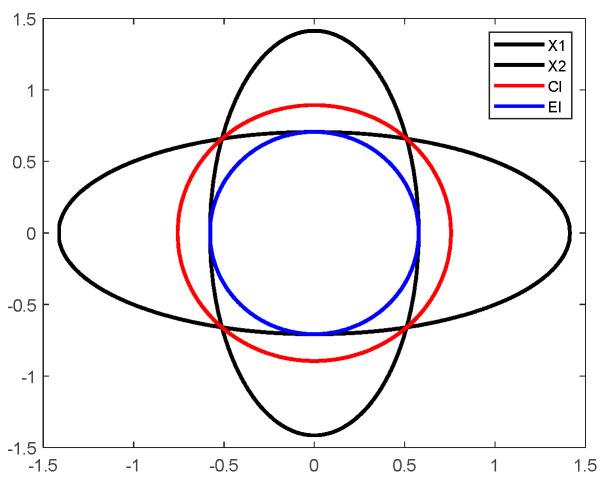
The fusion results of the two fusion methods for ellipsoids $X_{1}$ and $X_{2}$.

In order to verify the superior performance of the EI fusion method, we compare the differences of the two fusion methods by a numerical example [31]. Suppose there exist two ellipsoids $X_{1}$ and $X_{2}$, both of which have center 0 and shape matrices $P_{1}=diag\left(2,\frac{1}{2}\right)$ and $P_{1}=diag\left(\frac{1}{3},2\right)$, respectively. The fusion results of the two fusion methods for ellipsoids $X_{1}$ and $X_{2}$ are shown in Figure 3. The red ellipse indicates the fusion result of the CI method. The blue ellipse indicates the fusion result of the EI method. The CI fusion result contains an area larger than the intersection area of ellipsoids $X_{1}$ and $X_{2}$, which makes the fusion result too conservative. The EI fusion result is contained within the intersection area of ellipsoids $X_{1}$ and $X_{2}$, which ensures the accuracy of the fusion result.

Based on the described analysis, for the design of a distributed fusion estimator, it is a proper choice to use the EI fusion approach for the fusion estimator of the multi-source localization system [31]. Therefore, we design a DSEI fusion estimator in the fusion center. The fusion process is shown as follows:

$$x_{s,k}^{0}={\hat{x}}_{1,k},P_{s,k}^{0}=P_{1,k}$$

$$x_{s,k}^{i}=P_{s,k}^{i}({\left(P_{s,k}^{i-1}\right)}^{-1}x_{s,k}^{i-1}+P_{i+1,k}^{-1}{\hat{x}}_{i,k}-\Gamma_{i}^{-1}\gamma_{i})$$

$$P_{s,k}^{i}={\left({\left(P_{s,k}^{i-1}\right)}^{-1}+P_{i+1,k}^{-1}-\Gamma_{i}^{-1}\right)}^{-1}$$

$$\Gamma_{i}=S_{s,k}^{i-1}{\left(D_{s,k}^{i-1}\right)}^{-1/2}S_{i+1,k}D_{\Gamma_{i}}S_{i+1,k}^{-1}{\left(D_{s,k}^{i-1}\right)}^{1/2}{\left(S_{s,k}^{i-1}\right)}^{-1}$$

(27) $$\begin{array}{ll} \gamma_{i}= & \left(\left(P_{i+1,k}^{-1}-\Gamma_{i}^{-1}+\eta_{i}I\right)x_{s,k}^{i-1}+\left({\left(P_{s,k}^{i-1}\right)}^{-1}-\Gamma_{i}{}^{-1}+\eta_{i}I\right){\hat{x}}_{i+1,k}\right)\\ & \times {\left({\left(P_{s,k}^{i-1}\right)}^{-1}+{\hat{P}}_{i+1,k}^{-1}-2\Gamma_{i}^{-1}+2\eta_{i}I\right)}^{-1} \end{array}$$

Algorithm 1 summarizes the designed DSEI fusion estimation. The ellipsoidal center of the DSEI fusion estimation result is ${\hat{x}}_{k}=x_{s,k}^{L-1}$, and the shape matrix is $P_{k}=P_{s,k}^{L-1}$.

**Algorithm 1.** DSEI Fusion Estimator.**Input:** Estimated ellipsoids $\varepsilon_{i,k}\left({\hat{x}}_{i,k},P_{i,k}\right)$ for L local estimators; Number of sensor nodes L.**Output:** Initialization.
$$x_{s,k}^{0}={\hat{x}}_{1,k},P_{s,k}^{0}=P_{1,k}$$

1:Set i=1;2:Calculate the mean $\gamma_{i}$ and variance $\Gamma_{i}$ of the correlation information between the ellipsoid $\varepsilon_{s,k}^{i-1}$ and the ellipsoid $\varepsilon_{i+1,k}$;3:Calculate the fusion estimation of ellipsoid $\varepsilon_{s,k}^{i}\left(x_{s,k}^{i},P_{s,k}^{i}\right)$;4:Repeat steps 2 to 3 for the next sample


To verify whether the designed DSEI fusion estimator satisfies the consistency property, we perform consistency analysis on the designed fusion estimator. Based on the analysis of the fusion process of the previous numerical example, we can conclude that the shape matrix of the fusion result is smaller than the shape matrix of each local estimate [31]. That is, $tr\left(P_{s,k}^{1}\right)\leq tr\left(P_{s,k}^{0}\right),\;tr\left(P_{s,k}^{1}\right)\leq tr\left(P_{2,k}\right)$. 

Then, the iteration-based property analysis yields the results of the fusion of the first three local estimators as follows:

(28) $$tr\left(P_{s,k}^{2}\right)\leq tr\left(P_{s,k}^{1}\right),\;tr\left(P_{s,k}^{2}\right)\leq tr\left(P_{1,k}\right)$$

The collation gives:

(29) $$tr\left(P_{s,k}^{2}\right)\leq tr\left(P_{i,k}\right),i=1,2,3$$

As a total of *L* sensors are used to observe the system for the multi-source localization system, *L* local estimation results are obtained. The DSEI fusion estimator performs the EI fusion process *L* − 1 times.

(30) $$tr\left(P_{s,k}^{L-1}\right)\leq tr\left(P_{i,k}\right),i=1,2,\cdots ,L$$

In summary, our designed the DSEI fusion estimator outperforms the individual local estimators and provides a great guarantee for accurate localization results of the multi-source localization system.

## 5. Simulation Verification of Multi-Source Nonlinear Positioning System

To demonstrate the reliability of the designed DSEI fusion estimator based on the DSM filtering method, we consider a multi-source nonlinear localization system to validate the localization results of intelligent vehicles. The state-space model of the multi-source nonlinear localization system is shown as follows:

$$x_{k+1}=f_{k}\left(x_{k},u_{k}\right)+w_{k}$$

where the state vector for target tracking is denoted as $x_{k}={\left[p_{k}^{x},p_{k}^{y},v_{k},\theta_{k}\right]}^{T}$. $p_{k}^{x},p_{k}^{y}$ denotes the position of the smart car in the *X*-*Y* axis plane. $v_{k}$ denotes the current velocity of the smart car. $\theta_{k}$ denotes the magnitude of the wheel-to-body deflection angle of the smart car. The nonlinear state function $f_{k}$ is described by a state vector $x_{k}$ and an external control vector $u_{k}$, which is given as:

$$f_{k}\left(x_{k},u_{k}\right)=\left[\begin{array}{c} p_{k}^{x}+T_{0}\left(\nu_{k}+T_{0}u_{k}^{p}\right)\cos\left(\theta_{k}+T_{0}u_{k}^{r}\right)\\ p_{k}^{y}+T_{0}\left(\nu_{k}+T_{0}u_{k}^{p}\right)\sin\left(\theta_{k}+T_{0}u_{k}^{r}\right)\\ \nu_{k}+T_{0}u_{k}^{p}\\ \theta_{k}+T_{0}u_{k}^{r} \end{array}\right]$$

where the external control vector is defined as $u_{k}=\left[u_{k}^{p},u_{k}^{r}\right]$, $u_{k}^{p}$ = 0.85 indicates how fast the speed of the smart car changes, and $u_{k}^{r}$ = 0.15 describes how fast the deflection angle of the smart car changes. 

The center of the initial target tracking state ellipsoid is $x_{0}={\left[\begin{array}{cccc} 50 & 30 & 5 & 5 \end{array}\right]}^{T}$ and the shape matrix is $P_{0}=diag\left(200,200,200,200\right)$. Three sensors measure the position and angle to observe the smart vehicle. The nonlinear measurement equation is defined as follows:

$$y_{i,k}=h_{i,k}\left(x_{k}\right)+v_{i,k}$$

The position information $\left(s_{i}^{x},s_{i}^{y}\right)$ of three of the sensors is (100,150), (150,150), and (200,200). The nonlinear measurement function $h_{i,k}\left(x_{k}\right)$ is expressed as:

$$h_{i,k}\left(x_{k}\right)=\left[\begin{array}{c} \sqrt{{\left(p_{k}^{x}-s_{i}^{x}\right)}^{2}+{\left(p_{k}^{y}-s_{i}^{y}\right)}^{2}}\\ \theta_{k}-\arctan\left(\frac{p_{k}^{y}-s_{i}^{y}}{p_{k}^{x}-s_{i}^{x}}\right) \end{array}\right]$$

By solving the inverse of the measurement equation as required in (9), the following results are obtained:

$$P_{i,x}x_{i,k}≜\left[\begin{array}{l} \left(y_{i,k}^{1}-v_{i,k}^{1}\right)\cos\left(\theta_{k}-v_{i,k}^{2}\right)+s_{i}^{x}\\ \left(y_{i,k}^{1}-v_{i,k}^{1}\right)\sin\left(\theta_{k}-v_{i,k}^{2}\right)+s_{i}^{y} \end{array}\right]$$

where the transformation projection matrix $P_{i,x}$ is as follows:

$$P_{i,x}=\left[\begin{array}{cccc} \begin{array}{c} 1\\ 0 \end{array} & \begin{array}{c} 0\\ 1 \end{array} & \begin{array}{c} 0\\ 0 \end{array} & \begin{array}{c} 0\\ 0 \end{array} \end{array}\right]$$

The shape matrices of the ellipsoid containing the process noise $w_{k}$ and the measurement noise $v_{i,k}$ are:

$$Q_{k}=\mathrm{diag}(10,10,10,10)$$

$$R_{i,k}=\mathrm{diag}(100,0.5)$$

**Figure 4:**
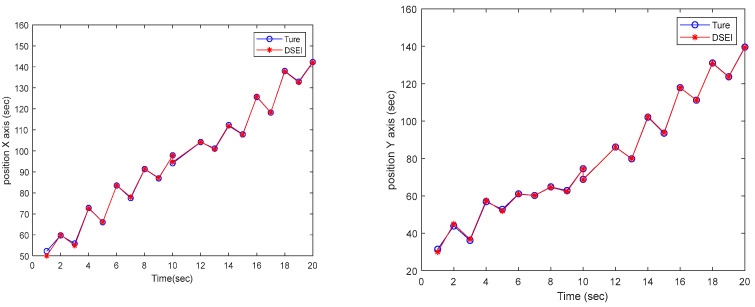
The target tracking performance of the DSEI fusion estimator in the *X*-axis and *Y*-axis directions.

**Figure 5:**
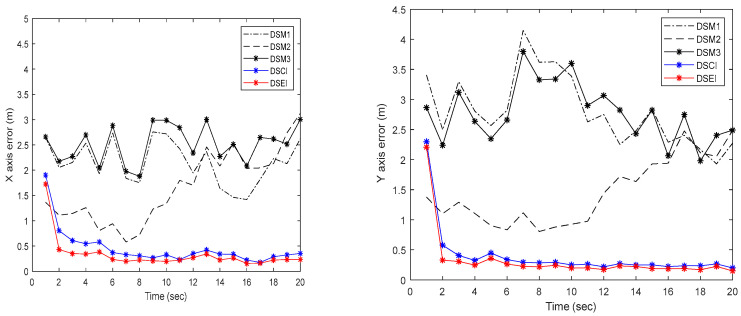
Comparison of errors in target tracking of positioning systems in the *X*-axis and *Y*-axis directions.

Based on the designed simulation example, we verify the tracking performance of the proposed DSEI fusion estimator with DSM filtering for the target’s state. The target tracking performance results are shown in Figure 4. We depict the position information in the *X*-axis and *Y*-axis directions, respectively. The results show that our designed DSEI fusion estimator using DSM filtering has good tracking performance. Meanwhile, to verify the superior performance of the fusion estimator, we compare the errors of the DSM filter estimation results and the fusion estimation results by comparing them. The results of the error analysis are shown in Figure 5. The results show that the fusion estimation error results are smaller than each DSM filter estimation error result, and the errors of the DSEI fusion estimation results are less than 0.5 m, which ensures the accuracy of the localization results.

**Figure 6:**
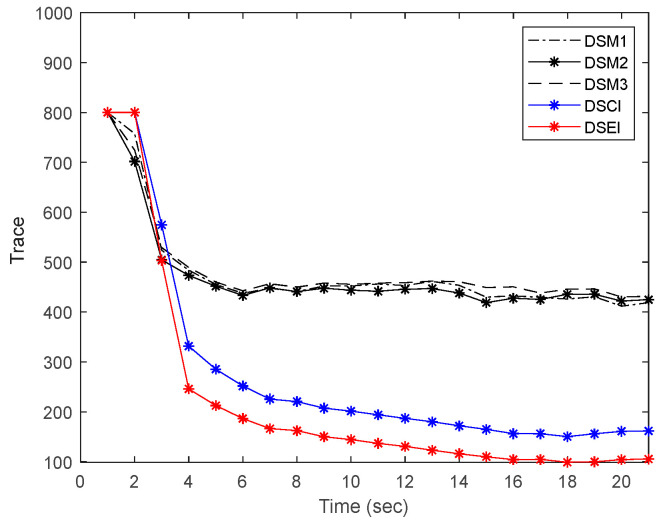
Comparison of the estimated ellipsoid size of the local DSM estimator with the DSCI and DSEI estimators.

In order to visualize the performance of the designed distributed fusion estimation, we compare the traces of the shape matrix of each estimation result. The comparison of the traces of the shape rectangles of each part of the ellipsoid is shown in Figure 6. The results show that the size of each estimated ellipsoid can converge quickly to a stable value. The reason is that the adopted DSM method can deal well with the semi-infinite optimization problem that occurs in nonlinear systems. Meanwhile, the sizes of the fused estimated ellipsoids at each moment are smaller than each DSM filter estimate. The results validate that the designed distributed system has good consistency. We also compare the distributed sequential covariance intersection (DSCI) fusion and DSEI fusion estimators. The results show that the estimated ellipsoid region of the DSCI fusion is larger than that of the DSEI estimation ellipsoid. It is due to the fact that DCEI does not describe the correlation information between local estimators, which leads to overly conservative estimation results. The algorithm of DSEI extracts the correlation information between local estimates and complements the fusion process by the correlation information, which ensures the accuracy of the estimation results. The feasibility of the scheme that the estimation results with high accuracy can be obtained by using the DSEI fusion estimator is also verified.

**Figure 7:**
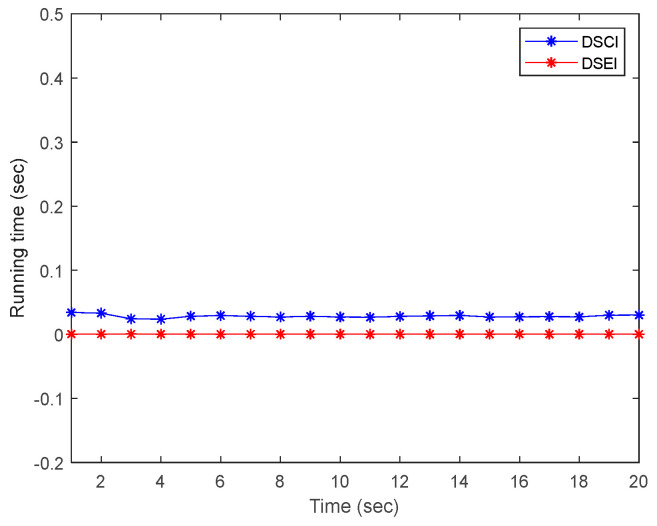
Comparison of the operation time of DSCI and DSEI estimators.

Meanwhile, in the simulation experiments, we analyze the computational cost of the proposed DSEI fusion algorithm. A comparison of the computational time cost of the DSCI fusion method and the DSEI fusion method during operation is shown in Figure 7. The results show that the computational cost used by the DSEI fusion estimator is smaller than that of the DSCI fusion estimator. The computational time required for each iteration of the DSEI fusion estimator is 0.002885 s, while that of the DSCI is 0.030756 s. It is due to the explicit description of the optimization problem in the DSEI algorithm. Explicit expressions will greatly reduce the computational cost reduction of the running process and can accelerate the output of the positioning results. Therefore, in terms of system performance and running time, our designed DSEI fusion estimator based on DSM filtering can guarantee the reliability and flexibility of the multi-source nonlinear localization system.

## 6. Conclusions

This paper addressed the reliability problem of target tracking for multi-source localization nonlinear systems. In order to obtain a flexible and accurate localization system, we proposed a fusion estimation method for multi-source localization nonlinear systems based on the fusion estimation of DSEI with the DSM filtering method. First, for the nonlinear systems with UAD noise characteristics, we used a dual method based on the first-order conditional gradient to solve the minimized ellipsoid containing the nonlinear function. Then, the information of the minimized ellipsoid was used to convert the nonlinear system into a linear system. Meanwhile, we used the principle of set-membership filtering to design a DSM local filter at the link nodes in the sensor network to filter out noise interference. To solve the situation that the correlation information is unknown due to the communication confusion between sensors, we designed a DSEI fusion estimation algorithm. It described the unknown correlation information between local estimates with explicit expressions. It performed sequential fusion at the fusion center. This method can obtain more accurate fusion results at a lower computational cost. The performance analysis demonstrated that the proposed fusion estimator can obtain estimation results with high accuracy and low computational complexity. In future research, we can apply the proposed method of DSEI fusion estimation based on the DSM filtering method to other practical applications such as smart grids.

## Data Availability

Not applicable.
